# Effectiveness of high-flow nasal cannula for tracheal intubation in the emergency department

**DOI:** 10.1186/s12873-022-00674-w

**Published:** 2022-06-23

**Authors:** Yumi Mitsuyama, Shunichiro Nakao, Junya Shimazaki, Hiroshi Ogura, Takeshi Shimazu

**Affiliations:** Department of Traumatology and Acute Critical Medicine, Osaka University Graduate School of Medicine, 2-15 Yamadaoka, Suita City, Osaka, 565-0871 Japan

**Keywords:** High-flow nasal cannula, Tracheal intubation, Emergency department

## Abstract

**Background:**

Tracheal intubation in the emergency department (ED) can cause serious complications. Available evidence on the use of a high-flow nasal cannula (HFNC) during intubation in the ED is limited. This study evaluated the effect of oxygen therapy by HFNC on oxygen desaturation during tracheal intubation in the ED.

**Methods:**

This was a single-center before-and-after study designed to compare two groups that received oxygen therapy during intubation: one received conventional oxygen, and the other received oxygen therapy using HFNC. We included non-trauma patients who required tracheal intubation in the ED. Linear regression analysis was performed to evaluate the relationship between oxygen therapy using HFNC and the lowest peripheral oxygen saturation (SpO_2_) during intubation in the conventional and HFNC groups.

**Results:**

The study population included 87 patients (conventional group, *n* = 67; HFNC group, *n* = 20). The median lowest SpO_2_ in the HFNC group was significantly higher than that in the conventional group (94% [84–99%] vs. 85% [76–91%], *p* = 0.006). The percentage of cases with oxygen desaturation to < 90% during the intubation procedure in the HFNC group was significantly lower than that in the conventional group (40% vs. 63.8%, *p* = 0.037). The use of HFNC was significantly associated with the lowest SpO_2_, and the use of HFNC increased the lowest SpO_2_ during intubation procedures by 3.658% (*p* = 0.048).

**Conclusion:**

We found that the use of HFNC during tracheal intubation was potentially associated with a higher lowest SpO_2_ during the procedure in comparison to conventional oxygen administration in non-trauma patients in the ED.

## Background

Tracheal intubation in the emergency department (ED) can increase the risk of adverse events and decrease the success rate of initial intubation due to limited patient history and examination [1]. In Japan, intubation in the ED is performed primarily by emergency medicine physicians and emergency medicine residents, and adverse events have been reported in 11% of cases [2, 3]. Mort reported that 2% of critically ill patients experienced cardiac arrest during emergency intubation, and peripheral oxygen saturation (SpO_2_) decreased below 70% in 83% of these cases [4]. The risk of hypoxemia is higher during emergency intubation in the ED, and the administration of oxygen during intubation is more important to avoid hypoxemia during the intubation procedure in comparison to during intubation in prepared settings [5].

Apneic oxygenation is the administration of oxygen in the absence of spontaneous respiration or mechanical ventilation to prevent desaturation during intubation [6]. Apneic oxygenation can prolong safe apnea time and reduce the risk of desaturation, thereby reducing hypoxia associated with intubation and increasing the safety of the intubation procedure [7]. The high-flow nasal cannula (HFNC) is used for the nasal delivery of heated and humidified oxygen at high flow rates. In recent years, there have been several reports of the use of HFNC in oxygen therapy during intubation maneuvers [8, 9]. The use of HFNC for apneic oxygenation during anesthesia induction was associated with longer safe apnea times and fewer adverse events than the usual methods such as face mask ventilation [10, 11].

The usefulness of apneic oxygenation with HFNC during intubation has been shown in patients in the intensive care unit (ICU) and operating room, but available evidence on the use of HFNC during intubation in the ED is limited [12]. Patients who require emergency intubation in the ED are often critically ill and unstable, and it may not be easy to predict a difficult intubation because of insufficient information on the patients. We hypothesized that apneic oxygenation with HFNC would be more useful than other methods because intubation in the ED may prolong safe apnea time. Thus, we aimed to evaluate the effect of oxygen administration by HFNC on oxygen desaturation during tracheal intubation in non-trauma patients who required tracheal intubation in the ED.

## Methods

### Study design

This was a single-center, prospective, observational study with before-and-after comparisons. This study was approved by the institutional review board of Osaka University (approval no. 19142). Because all data for this study were obtained from patient medical records, the requirement for consent from individual patients was waived.

### Setting

This study was performed at the Trauma and Acute Critical Care Center of Osaka University Hospital in Osaka, Japan. Our center is a tertiary care facility that receives only critically ill patients; about 1100 patients are transported to the center annually. The ED is equipped with two resuscitation rooms and one angiography room. Patients requiring hospitalization are admitted to the ICU attached to the center and are managed by dedicated intensive care physicians in the same department. For tracheal intubation, the team consists of one attending physician and at least two nurses. When an emergency medicine resident performs tracheal intubation, the team consists of the resident, an attending physician, and at least two nurses. The staff members are familiar with the use of HFNC. The study period was 1 year and 5 months, from October 2018 to March 2020. From October 2018 to September 2019, tracheal intubation was performed under conventional oxygen administration (conventional group), and from October 2019 to March 2020, tracheal intubation was performed using HFNC (HFNC group). A one-week period from September 24 to 30, 2019, was set as a training period to familiarize the medical staff with the HFNC procedure.

### Participants

We included non-trauma patients of ≥ 18 years of age who required tracheal intubation regardless of having hypoxia and who were treated in the ED (i.e., those who required tracheal intubation for airway protection were included in our study). Patients with non-traumatic cardiopulmonary arrest at the time of admission or who had a do-not-resuscitate order were excluded from the study. Furthermore, patients whose records were missing SpO_2_ data and patients in the HFNC group in whom HFNC was not used because equipment was unavailable were excluded from the analysis.

### Procedures

In the conventional group, passive oxygenation and positive pressure ventilation using a bag-valve mask with a reservoir bag were provided from the time when tracheal intubation was decided. Oxygen was administered at a rate of 10 L/min. Sedatives, analgesics, and neuromuscular blocking agents were administered at the discretion of the emergency physicians. In the HFNC group, a nasal interface (Optiflow™, Fisher & Paykel Healthcare, Auckland, New Zealand) connected to HFNC (Airvo^TM^2, Fisher & Paykel Healthcare, Auckland, New Zealand) was attached to the patient from the time when tracheal intubation was decided. The inspiratory fraction of oxygen (FiO_2_) was set to 1.0 with a flow of 60 L/min. The gas was heated to 37 °C and humidified. The HFNC was used continuously until the tracheal tube was connected to the ventilator. Intubation equipment, sedatives, analgesics, neuromuscular blocking agents, and intubation maneuvers were performed according to the usual methods. All tracheal intubations in the ED were performed by board-certified emergency medicine physicians (post-graduate year > 5) and emergency medicine residents (post-graduate year 3 to 5) who had completed training in airway management. Intubation was performed using a Macintosh laryngoscope or McGRATH™ MAC video laryngoscope (Medtronic, Minneapolis, MN). We used medications for intubation recommended by the Japanese Society of Anesthesiologist [13]. The following medications were available for tracheal intubation in the ED: propofol, midazolam and ketamine (as sedatives), fentanyl (as an analgesic), and rocuronium (as a neuromuscular blocking agent).

### Variables

Demographic variables included age, sex, and body mass index. The clinical data collected in the ED included SpO_2_, heart rate, systolic blood pressure, respiratory rate, and Glasgow Coma Scale. Arterial blood gasses were collected immediately after arrival at the ED. The severity of the patient’s illness on admission was assessed by the Sequential Organ Failure Assessment (SOFA) score and the Acute Physiologic Assessment and Chronic Health Evaluation (APACHE) II score. The initial diagnosis on admission was noted according to the medical records. Septic shock was defined according to the Sepsis-3 criteria, and hemorrhagic shock was defined as shock with a systolic blood pressure of < 90 mmHg due to bleeding, such as gastrointestinal bleeding [14]. Cardiogenic shock was extracted from the emergency physician’s medical record. All data were collected by an independent researcher using a standardized data collection form based on information provided in the electronic medical record, in which SpO_2_ was automatically recorded every 3 s during tracheal intubation. The primary endpoint was the lowest SpO_2_ during the intubation procedure beginning from the administration of the induction drug to tracheal tube placement. In the ED, SpO_2_ was measured by a BluPRO SpO_2_ reusable sensor (Nihon Kohden, Tokyo, Japan) placed on a fingertip. The lowest SpO_2_ was defined as the lowest level of SpO_2_ measured during tracheal intubation. The secondary outcome was the percentage of cases with SpO_2_ < 90% during the intubation procedure. Length of mechanical ventilation, length of ICU stay, ICU mortality, and 28-day mortality were also evaluated.

### Sample size

The sample size was estimated based on previous studies, assuming that the standard deviation of the lowest SpO_2_ was 12% and that the use of HFNC would improve the lowest SpO_2_ by 5% over conventional oxygen administration [15]. We calculated an alpha error of 0.05 and a power of 0.8, assuming a dropout rate of 10% (e.g., missing data), and expected to enroll 100 patients in each group. However, enrollment was terminated mid-study due to restrictions on the use of HFNC in the ED after March 2020 due to the novel coronavirus infection epidemic. Termination was for administrative reasons. Hospital administrators banned the use of HFNC in our ED due to concerns about aerosol generation by the use of HFNC on patients with the novel coronavirus disease. 

### Statistical analysis

Continuous variables are shown as the median and interquartile range (IQR), and categorical variables are shown as frequencies and percentages. The Wilcoxon rank-sum test was used to test continuous variables, and Fisher’s exact test was used to test the nominal variables. A linear regression analysis, with adjustment for age, use of neuromuscular blocking agents, intubation by emergency medicine residents, and APACHE II score, was performed to evaluate the relationship between the use of HFNC and the lowest SpO_2_ during intubation in the conventional and HFNC groups. All analyses were performed using JMP 15 (SAS Institute Inc., Cary, NC, USA).

## Results

**Fig. 1:**
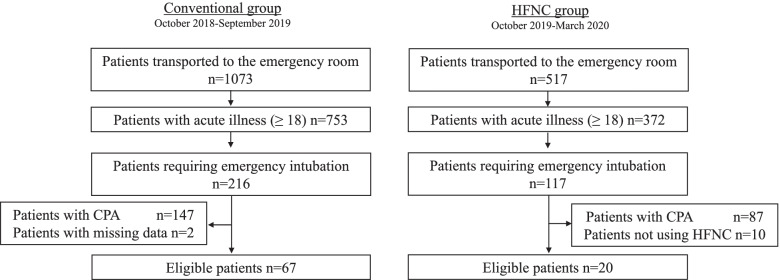
Patient flow in the present study. *CPA* cardiopulmonary arrest, *HFNC* high-flow nasal cannula

Figure 1 shows a flow diagram of the present study. Although we planned to exclude those patients with do-not-resuscitate order, no patients were excluded because of it. Sixty-seven patients in the conventional group and 20 patients in the HFNC group were included in the analysis.

Table 1 shows the patient characteristics of each group. The median initial SpO_2_ was 96% (IQR 83–99%) in the HFNC group and 94% (IQR 84–99%) in the conventional group. The median Glasgow Coma Scale was 13 (IQR 7–15) in the HFNC group and 9 (IQR: 6–14) in the conventional group. The APACHE II score of the conventional group was significantly higher than that of the HFNC group (17 [IQR 8–24] vs. 24 [IQR 17–27], *p* = 0.007). In both groups, the most frequent diagnosis was stroke, followed by pneumonia. The most frequent indication for intubation was consciousness disorder (50.0% in the HFNC group and 50.7% in the conventional group), followed by hypoxemia (30.0% in the HFNC group and 25.4% in the conventional group). In both groups, most patients were intubated using sedatives (90.0% in the HFNC group and 85.1% in the conventional group), and neuromuscular blocking agents (80.0% in the HFNC group and 67.2% in the conventional group). The number of intubations performed by emergency medicine residents was higher in the HFNC group than that in the conventional group. No intubations were performed by residents of post-graduate year < 2. There were no occurrences of cardiac arrest during or after intubation.

**Table 1:** Patient characteristics

	HFNC group		Conventional group		*p* value
Variables	*n* = 20		*n* = 67		
Age, years, median (IQR)	65	(49–77)	70	(61–81)	0.184
Male, n (%)	12	(60.0)	35	(52.2)	0.543
BMI, median (IQR)	20.5	(18.2–23.3)	21.5	(19.2–23.5)	0.361
Vital signs on arrival, median (IQR)					
SpO_2_, %	96	(83–99)	94	(84–99)	0.816
Respiratory rate, breaths/min	20	(15–25)	22	(16–26)	0.282
Systolic blood pressure, mmHg	135	(110–176)	128	(101–146)	0.300
Heart rate, beats/min	110	(80–133)	103	(89–112)	0.320
Glasgow coma scale, median (IQR)	13	(7–15)	9	(6–14)	0.097
Arterial blood gas analysis, median (IQR)					
pH	7.38	(7.32–7.44)	7.37	(7.23–7.44)	0.664
PaO_2,_ mmHg	83	(56.2–209.8)	77	(50.6–123)	0.294
PaCO_2,_ mmHg	40.5	(34.7–46.0)	37.7	(29.5–48.3)	0.244
SOFA score, median (IQR)	7	(4–9)	8	(6–10)	0.112
APACHE II score, median (IQR)	17	(8–24)	24	(17–27)	0.007
Initial diagnoses, n (%)					
Stroke, n (%)	9	(45.0)	28	(41.8)	0.803
Pneumonia, n (%)	4	(20.0)	13	(19.4)	0.953
Shock, n (%)					
Septic shock, n (%)	1	(5.0)	8	(11.9)	0.678
Hemorrhagic shock, n (%)	2	(10.0)	2	(3.0)	0.225
Cardiogenic shock, n (%)	0	(0)	2	(3.0)	0.437
Acute heart failure, n (%)	1	(5.0)	5	(7.5)	0.705
Acute renal failure, n (%)	0	(0)	4	(6.0)	0.570
Chronic obstructive pulmonary disease, n (%)	1	(5.0)	1	(1.5)	0.409
Others, n (%)	2	(10.0)	1	(1.5)	0.812
Indication for intubation					
Consciousness disorder, n (%)	10	(50.0)	34	(50.7)	0.954
Hypoxemia, n (%)	6	(30.0)	17	(25.4)	0.774
Emergency operation, n (%)	3	(15.0)	5	(7.5)	0.378
Shock, n (%)	3	(15.0)	10	(14.9)	0.994
Drugs used for intubation					
With sedatives (Propofol, midazoram), n (%)	17	(85.0)	54	(80.6)	0.657
Other sedatives, n (%)	1	(5.0)	3	(4.5)	0.923
No sedatives, n (%)	2	(10.0)	10	(14.9)	0.725
Analgesic, n (%)	12	(60.0)	42	(62.7)	0.405
Neuromuscular blocking agent, n (%)	16	(80.0)	45	(67.2)	0.829
Intubation by emergency medicine residents	13	(65.0)	24	(35.8)	0.038

*BMI* Body mass index, *SpO*_*2*_ Saturation of percutaneous oxygen, *SOFA score* Sequential Organ Failure Assessment score, *APACHE II score* Acute Physiologic Assessment and Chronic Health Evaluation

Table 2 shows the outcomes. The median lowest SpO_2_ in the HFNC group was significantly higher than that in the conventional group (94% [IQR 84–99%] vs. 85% [IQR 76–91%], *p* = 0.006). The median length of mechanical ventilation in the HFNC group was significantly shorter than that in the conventional group (3 days [IQR 2–6 days] vs. 7 days [IQR 3–11 days], *p* = 0.006). The median length of ICU stay in the HFNC group was significantly shorter than that in the conventional group (7 days [IQR 4–15 days] vs. 13 days [IQR 8–19 days], *p* = 0.038). The percentage of cases with oxygen desaturation to < 90% during the intubation procedure in the HFNC group was significantly lower than that in the conventional group (8/20 [40%] vs. 44/67 [63.8%], *p* = 0.037).

**Table 2:** Outcomes including lowest SpO_2_ between the HFNC group and the conventional group

	HFNC group		Conventional group		*p* value
Variables	*n* = 20		*n* = 67		
Lowest SpO_2_(%), median (IQR)	94	(84–99)	85	(76–91)	0.006
Oxygen desaturation to < 90% during intubation procedure, n (%)	8	(40.0)	44	(63.8)	0.037
Length of mechanical ventilation, days, median (IQR)	3	(2–6)	7	(3–11)	0.006
Length of ICU stay, days, median (IQR)	7	(4–15)	13	(8–19)	0.038
ICU mortality	2	(10.0)	5	(7.5)	0.658
28-day mortality, n (%)	2	(10.0)	6	(9.0)	0.888

*HFNC* High-flow nasal cannula, *ICU* Intensive care unit

The results of the multiple regression analysis are shown in Table 3. We used the following five factors as covariates: HFNC, age, use of neuromuscular blocking agents, intubation by emergency medicine residents, and APACHE II score. The lowest SpO_2_ in the HFNC group was significantly higher than that in the conventional group (estimated difference 3.658%, 95% confidence interval 0.031–7.285%, *p* = 0.048).

**Table 3:** Multiple linear regression analysis comparing the lowest SpO_2_ between the HFNC group and the conventional group

Term	Coefficient	Standard Error	Lower 95%	Upper 95%	*p* value
HFNC	3.658	1.823	0.031	7.285	0.048
Age	-0.126	0.099	-0.323	0.071	0.207
Neuromuscular blockade	-2.117	1.543	-5.188	0.954	0.174
Intubation by emergency medicine residents	-0.773	1.480	-3.719	2.173	0.603
APACHE II score	0.073	0.189	-0.303	0.450	0.699

*R*.^*2*^ = 0.12

*HFNC* High-flow nasal cannula, *APACHE II score* Acute Physiologic Assessment and Chronic Health Evaluation

## Discussion

We showed that the use of HFNC increased the lowest SpO_2_ during the intubation procedure in non-trauma patients who required tracheal intubation in the ED. The use of intermittent oxygenation with HFNC may have reduced the occurrence of hypoxemia in non-trauma patients who required tracheal intubation in the ED and made the intubation procedure safer.

Guitton et al. reported that the use of HFNC during intubation in 184 patients with non-severe hypoxemia in the ICU did not result in a significant difference in the lowest SpO_2_ during the intubation procedure in comparison to the administration of conventional oxygen; however, it resulted in fewer instances of desaturation to < 95% in the HFNC group [11]. The success rate of initial intubation may be lower than that in the ICU or operating room [16]. Repeated intubation procedures may increase adverse events such as hypoxemia [17]. The use of HFNC in apneic oxygenation can result in an increase of the lowest SpO_2_ during the intubation procedure, which suggests that its use may prolong safe apnea time and could decrease adverse events. The reduced risk of desaturation allows clinicians to avoid having to prematurely abandon an ongoing intubation, providing adequate time to perform tracheal intubation and focus on the necessary maneuvers [18]. The nasal cannula used for HFNC does not interfere with the wearing of a face mask when additional mask ventilation is required. In the ED setting, the use of HFNC with prolonged apnea times may be advantageous for patients with known or unknown intubation difficulties.

In the present study, there were significant differences in the duration of mechanical ventilation and the length of ICU stay between the two groups. Although Sakles et al. reported delayed complications, such as respiratory tract infection, after emergency intubation, it is difficult to evaluate the relationship between the lowest SpO_2_ during an intubation procedure and the length of mechanical ventilation and length of ICU stay [19]. The APACHE II score was significantly higher and the Glasgow Coma Scale was lower in the conventional group than in the HFNC group, which may be because the conventional group included more critically ill patients.

### Limitations

The present study was associated with some limitations. First, the number of cases was relatively small because the study had to be interrupted due to the novel coronavirus disease outbreak, which occurred during the study period. This interruption may have resulted in seasonal variations in the number of patients. Although it seemed that the patients in the conventional group were sicker, severity was adjusted for in a multiple regression analysis, and the results were consistent. Second, because this was a before-and-after study, there may be unadjusted cofounding factors, such as devices and drugs used for intubation and the skill level of the physicians. However, there was no change in available devices or medications used for intubation, nor was there a change in staff members. To minimize potential confounding factors, we performed a multivariable analysis, and the results from the univariable and multivariable analyses were consistent. Third, the technique of the physician who performed the intubation was not analyzed. Cook et al. reported immaturity of the physician's technique was a risk factor for intubation failure and adverse events [20]. However, as all tracheal intubations in this study were performed by experienced emergency physicians, we believe this potential bias was minimal. Fourth, some cases were excluded from this study. Because of the limited numbers of HFNC available in the ED, there were cases in the HFNC group for which HFNC could not be used. Furthermore, information on the time from preparation for intubation to the completion of intubation was not collected. Although a longer time to complete intubation may increase the risk of hypoxia, we could not assess the difference in intubation procedure times between the groups. Finally, as we did not collect information on the assessment of difficult intubation, we were not able to adjust for the related factors.

## Conclusions

We found that the use of HFNC during tracheal intubation was potentially associated with a higher lowest SpO_2_ during the procedure than conventional oxygen administration in non-trauma patients in the ED. Our results suggest that the use of HFNC during tracheal intubation could be helpful for safer intubation in the ED.

## Data Availability

The datasets used and/or analyzed during the current study are available from the corresponding author on reasonable request.

## Abbreviations

ED: Emergency department
SpO_2_: Peripheral oxygen saturation
HFNC: High-flow nasal cannula
ICU: Intensive care unit
FiO_2_: Inspiratory fraction of oxygen
SOFA: Sequential Organ Failure Assessment
APACHE: Acute Physiologic Assessment and Chronic Health Evaluation
IQR: Interquartile range
